# Enhanced diagnostic precision of colorectal sessile lesions with blue laser imaging and JNET classification: a multicenter retrospective study in Chinese cohorts

**DOI:** 10.3389/fonc.2025.1534922

**Published:** 2025-05-30

**Authors:** Feng Wu, Wenxin Tan, Panpan Liu, Weiguang Qiao, Tongyin Xing

**Affiliations:** ^1^ Department of Critical Care Medicine, Nanfang Hospital, Southern Medical University, Guangzhou, China; ^2^ Department of Gastroenterology, Nanfang Hospital, Southern Medical University, Guangzhou, China

**Keywords:** blue laser imaging (BLI), colorectal cancer, colorectal sessile lesions, pathological prediction, Japan NBI Expert Team (JNET) classification

## Abstract

**Background:**

Colorectal cancer (CRC), a leading global malignancy, underscores the need for precise endoscopic diagnosis. Blue Laser Imaging (BLI), a novel endoscopic technology enhancing mucosal surface visualization, combined with the Japan NBI Expert Team (JNET) classification, has shown promise in characterizing colorectal lesions. However, its diagnostic performance in Chinese populations and the impact of endoscopist experience remain underexplored.

**Methods:**

In this multicenter, retrospective study, 131 colorectal sessile lesions were enrolled. The lesions’ characteristics were assessed by both expert and trainee endoscopists, utilizing magnified BLI in combination with the JNET classification system to establish diagnostic predictions. This approach allowed for a comparative evaluation of diagnostic accuracy between experienced and less experienced practitioners.

**Results:**

Pathological diagnoses confirmed 2 hyperplastic/sessile serrated lesions (HP/SSL), and 70 low-grade dysplasia (LGD) among the 131 lesions. There were 36 high-grade dysplasia (HGD), 16 superficial submucosal invasive cancers (m-SMs), and 7 deep submucosal invasive cancers (SM-d) demonstrated. The performance metrics for expert and trainee endoscopists in evaluating JNET type 2A(LGD) were as follows: expert endoscopists demonstrated a sensitivity, specificity, positive predictive value (PPV), negative predictive value (NPV), and accuracy of 93%, 93.3%, 94.3%, 91.8%, and 93.1%, respectively; trainee endoscopists showed a sensitivity, specificity, PPV, NPV, and accuracy of 64.6%, 63.5%, 72.9%, 54.1%, and 64.1%, respectively(p<0.01). For JNET type 2B(HGD/m-SMs), expert endoscopists exhibited a sensitivity, specificity, PPV, NPV, and accuracy of 88.2%, 91.3%, 86.5%, 92.4%, and 90.1%, respectively; trainee endoscopists showed a sensitivity, specificity, PPV, NPV, and accuracy of 59.6%, 73.4%, 73.4%, and 67.9%, respectively(p<0.01).

**Conclusions:**

BLI-JNET provides high diagnostic accuracy for colorectal sessile lesions in expert endoscopists, validating its clinical utility. However, trainee endoscopists exhibited significantly low accuracy, underscoring the need for structured training. The proportion of HGD/m-SMs in JNET type 2B lesions within the Chinese cohort (88.2%) was significantly higher than that reported in Japanese data (Kobayashi et al., 2019), highlighting the need to optimize classification systems by incorporating region-specific characteristics.

## Introduction

Colorectal cancer (CRC) is the third most common malignancy globally, with over 1.9 million new cases and 900,000 deaths reported in 2022 (1). CRC typically develops from precancerous lesions including sessile serrated lesions (SSLs) and adenomas through long-term progression, primarily via two carcinogenic pathways: the ‘serrated pathway’ and the ‘adenoma-carcinoma sequence’ (2, 3). Molecular studies reveal that approximately 30% of CRC cases are associated with the serrated pathway, with characteristics of BRAF mutations, microsatellite instability (MSI), and CpG island methylator phenotype (CIMP) (4). Early detection and resection of these lesions significantly reduce CRC mortality (5). Notably, early-stage CRC (Tis/T1a) with minimal lymph node metastasis risk has a possibility to be curatively treated by endoscopic resection, while T1b cancers require surgical intervention (6).

According to the Paris endoscopic classification, superficial colorectal lesions are categorized into elevated (type 0-I) and non-elevated (type 0-II) morphologies (7–9). Sessile lesions (0-Is) demonstrate substantially higher risks of submucosal invasion compared to pedunculated lesions (0-Ip), with deep submucosal invasion (SM-d) occurring in 15%-20% of cases (6, 8). Inaccurate identification may lead to incomplete resection or increased lymph node metastasis risks (6). SSLs, characterized by flat morphology (Paris IIa/Is type), indistinct margins, and predominant occurrence in the right colon, pose diagnostic challenges in endoscopic practice (10, 11). The WASP classification system developed by IJspeert et al. improves SSL detection accuracy (post-training diagnostic accuracy increases from 0.63 to 0.79) by integrating narrow-band imaging (NBI) with morphological features (10). However, conventional NBI demonstrates only 76.1% sensitivity in clinical practice, leaving room for improvement (12).

Blue light imaging (BLI), a novel endoscopic technology utilizing 410 nm laser light to enhance mucosal contrast, has shown potential in increasing colorectal lesion detection rates (13). When combined with the JNET classification system (Type 1: hyperplastic polyps/SSLs; Type 2A: low-grade dysplasia; Type 2B: high-grade dysplasia/superficial submucosal invasion; Type 3: deep submucosal invasion) (14), BLI demonstrates diagnostic efficacy comparable to NBI in predicting colorectal lesion characteristics (14). Nevertheless, SSLs remain critical targets for CRC prevention due to their molecular heterogeneity (e.g., BRAF mutations and CIMP-H) and substantial contribution to post-colonoscopy CRC (PCCRC), accounting for 18%-20% of cases (4, 15). Tan et al. reported an overall SSL prevalence of 4.0% in Asian populations, with linked color imaging (LCI) and cap-assisted colonoscopy improving detection rates by 63% and 75%, respectively (11).

Blue light imaging (BLI; Fujifilm Co.), utilizing 410 nm laser light to enhance mucosal contrast, has shown potential in increasing colorectal lesion detection rates (13). When combined with the JNET classification system (Type 1: hyperplastic polyps/SSLs; Type 2A: low-grade dysplasia; Type 2B: high-grade dysplasia/superficial submucosal invasion; Type 3: deep submucosal invasion) (14), BLI demonstrates comparable diagnostic efficacy to NBI in predicting colorectal lesion characteristics (14). Nevertheless, SSLs remain critical targets for CRC prevention due to their molecular heterogeneity (e.g., BRAF mutations and CIMP-H) and substantial contribution to post-colonoscopy CRC (PCCRC), accounting for 18%-20% of cases (4, 15). Tan et al. reported an overall SSL prevalence of 4.0% in Asian populations, utilizing linked color imaging (LCI) and cap-assisted colonoscopy improving detection rates by 63% and 75%, respectively (11).

Although the BLI-JNET classification has been validated for sessile lesions in Japanese populations (16), its applicability in Chinese cohorts remains unverified. Region-specific studies are warranted considering differences in genetic backgrounds (e.g., CRC molecular subtypes), environmental factors (e.g., dietary patterns), and medical practices (e.g., endoscopic training systems) (17). Furthermore, the impact of endoscopist experience on BLI-JNET diagnostic accuracy remains unquantified. Researchers like Ladabaum emphasize that SSL detection rate (SSLDR) should be prioritized as a quality control metric equivalent to adenoma detection rate (ADR) to optimize CRC prevention strategies (15). This pioneering study systematically evaluates the diagnostic performance of BLI-JNET for sessile lesions in Chinese populations and compares interpretation differences between trainee and expert endoscopists, aiming to: (i) Establish BLI-JNET diagnostic efficacy in Chinese cohorts; (ii)Elucidate mechanisms underlying endoscopist experience-related classification discrepancies. The findings will not only address China-specific data gaps but also provide new evidence for global CRC prevention strategy optimization.

## Materials and methods

### Study design and lesions

Four Chinese universities and academic centers contributed to this retrospective study. The patient cohort was selected based on the following criteria from January 1st, 2016, to December 31st, 2018: (i) individuals aged 18 years and older; (ii) newly diagnosed with 0-Is or 0-Isp lesions exceeding 1 cm in size; (iii) Lesions observed using blue light imaging (BLI) mode in combination with magnification; (iv) lesions evaluated with the JNET classification; (v) lesions resected via endoscopy. Exclusion criteria included: (i) unclear endoscopic images; (ii) observations using narrow-band imaging (NBI); (iii) indeterminate pathology; and (iv) absence of JNET classification for evaluating the surface. Ethical approval was obtained (ChiECRCT20190162), and the study was registered (ChiCTR1900025423).

The study utilized data from 2016 to 2018 to ensure uniformity in the first-generation Fuji BLI system (LASEREO model) across all participating centers (13, 18). Post-2018, some centers upgraded to the BLI-bright system, which differs in technical parameters (e.g., laser wavelength). Future studies will integrate multi-center data from newer systems to validate long-term outcomes. *A priori* power analysis (α=0.05, β=0.2) indicated that ≥120 lesions were required to detect≥20% diagnostic accuracy between expert and trainee endoscopists (19).

### Endoscopic procedures

Colonoscopies, conducted utilizing the Blue Light Imaging (BLI) system, either ELUXEO or LASEREO by Fujifilm Co., are paired with high-definition colonoscopies (EC-L590ZW, EC-L590ZP, and EC-760ZP-V/M models) from the same manufacturer. These systems enhance the visualization of superficial vessels and gastrointestinal mucosal structures. These advanced imaging technologies provide optimal illumination and image clarity to examine colorectal lesions in detail. The high-definition colonoscopies are known for their superior image quality and advanced features such as a wide viewing angle and large working channel diameter, which facilitate the detection and characterization of lesions. Six expert endoscopists, who were experienced in magnified NBI colonoscopies, performed the endoscopic submucosal dissections (ESDs). Comprehensive patient data, encompassing age, sex, lesion location, size, and morphology, were meticulously documented for thorough analysis.

### Histological diagnosis

All of the ESD specimens were fixed in 10% buffered formalin within a 24-hour window post-procedure to ensure optimal tissue preservation for histopathological assessment. The histological interpretation of the lesions was conducted in strict accordance with the World Health Organization (WHO) guidelines, ensuring standardization and reliability of the diagnostic process. Our evaluation of the histological samples was performed in a blinded manner, with no access to clinicopathologic or endoscopic data, thereby ensuring that the interpretations were solely based on the histological features of the samples. This approach is essential for upholding the objectivity and integrity of the histopathological evaluation process.

### Endoscopic image evaluation

**Figure 1:**
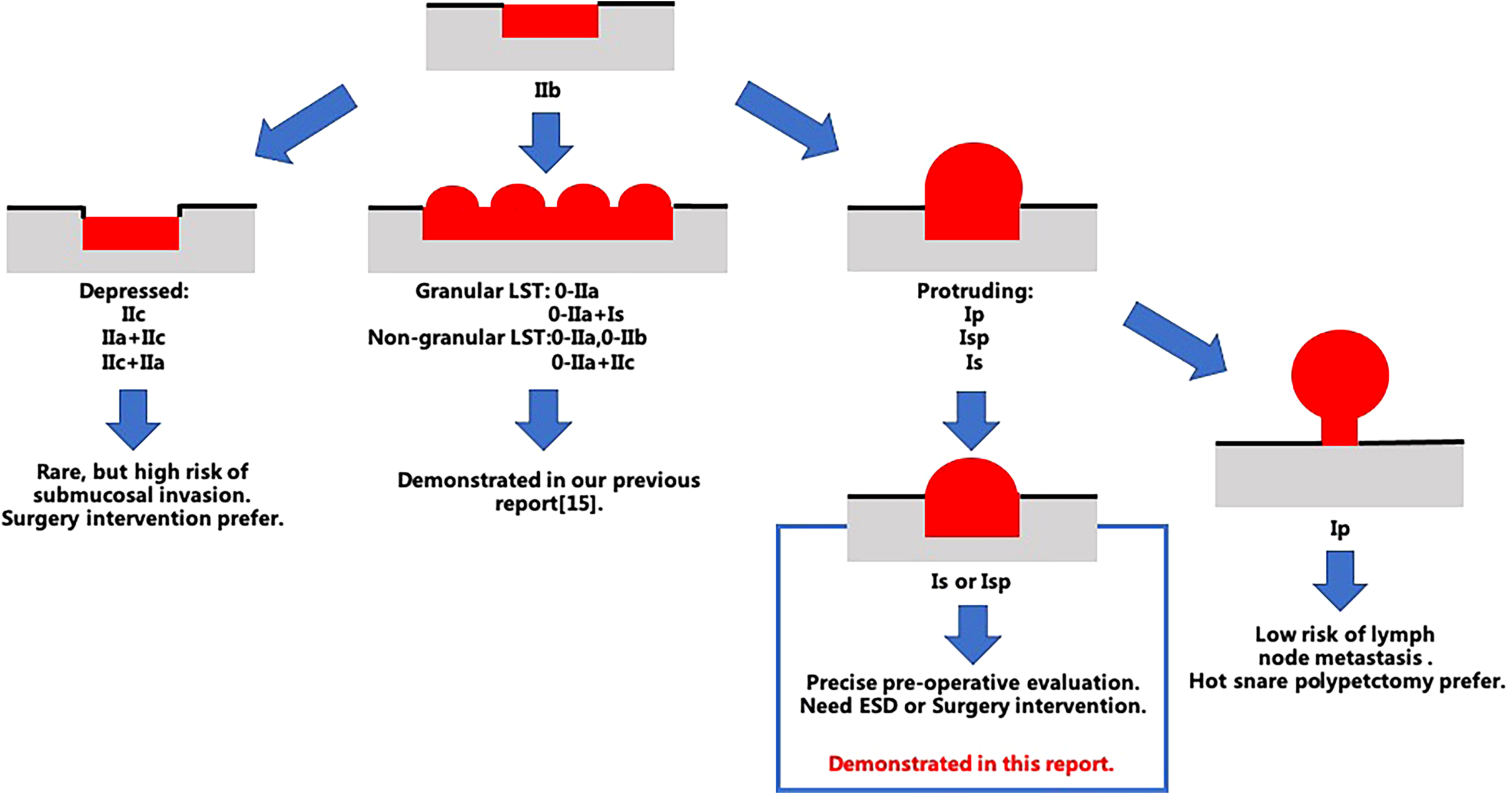
Schematic representation of the Paris classification for colorectal lesions.

Endoscopic image assessments were performed by a team comprising nine endoscopists, comprising three expert endoscopists and six trainee endoscopists. The expert endoscopists, who were adept in the JNET classification and BLI, possessed over three years of endoscopy experience and served as instructors at the JNBI workshop. In contrast, the trainee endoscopists, hailing from rural China with limited familiarity with the JNBI classification, received standard training on the classification system and BLI use during the JNBI workshop. The expert endoscopists had completed more than 100 BLI-magnified colonoscopies, while the trainee endoscopists had conducted fewer than 30. Utilizing BLI with magnification, both the vascular and surface patterns of lesions were scrutinized in accordance with the JNET classification system (**Figure 1**).

To maintain clinical and histopathological blindness among the endoscopists, four nurses independently collected the images of the colon lesions. Thereafter, three expert endoscopists assigned the JNET classification based on these images. In instances of discordance, a re-evaluation was initiated to establish a consensus.

### Statistical analysis

Continuous variables were expressed as the mean ± standard deviation and were analyzed by using a Student’s t-test for comparisons. And categorical variables were presented as percentages or proportions and were evaluated with the use of Pearson Chi-square test or Fisher exact test, depending on the data distribution. The sensitivity, specificity, positive predictive value (PPV), negative predictive value (NPV), and accuracy of the JNET classifications for both trainee and expert endoscopists were determined and compared against the histopathology of colorectal lesions, which served as the reference standard. The consistency between JNET classification and pathological outcomes of the ESD specimens was quantified using kappa coefficients. Statistical analyses were performed using SPSS software (version 20.0; SPSS, Chicago, IL). A two-tailed P-value less than 0.05 was considered statistically significant.

## Results

### Characteristics of eligible patients and lesions

The characteristics of the enrolled patients and colorectal lesion data were summarized in **Table 1**. Our study cohort comprised 131 patients, 38.2% of whom were female. We assessed 131 colorectal sessile lesions using magnified BLI and resected them with ESD procedures. The mean lesion size was 26.02 ± 11.20 mm (range 10.00-60.00 mm). Lesion distribution was as follows: rectum (64 cases, 48.9%), sigmoid colon (40 cases, 30.5%), transverse colon (12 cases, 9.2%), descending colon (8 cases, 6.1%), ascending colon (6 cases, 4.6%), and ileocecal junction (1 case, 0.8%).

**Table 1:** The characteristics of patients.

Characteristics	Total N. of lesions (%) 131(100)*
**Age, y** (Mean Std. Deviation)	56.15 + 11.95
Sex	
male	81 (61.8)
female	50 (38.2)
Location N. of lesion%	
Rectum	64 (48.9)
Sigmoid colon	40 (30.5)
Descending colon	8 (6.1)
Transverse colon	12 (9.2)
Ascending colon	6 (4.6)
ileocecal junction	1 (0.8)
Size (mm)	
Mean ± Std. Deviation	26.02 ± 11.20
(Range)	(10, 60)

### Diagnostic yield of magnified BLI with JNET classification for colorectal sessile lesions

Histological diagnoses of 131 sessile lesions were categorized as follows: 2 cases of HP/SSL, 70 of LGD, 36 of HGD, 16 of m-SMs, and 7 of the SM-d lesions (refer to **Table 2**). The correlation between histological findings and the JNET classification is detailed in **Table 2** for both expert and trainee endoscopists.

**Table 2:** The histologic findings and JNET classification between trainees and experts.

Group		Histologic findings				
					Carcinoma	
JNET classification	N (%)	HP or SSp/SSL	LGD	HGD	m-SMs	SMd
Trainee						
Type 2A	79 (100)	2 (2.5)	51(64.6)	16(20.3)	5 (6.3)	5 (6.3)
Type 2B	52 (100)		19(36.5)	20(38.5)	11(21.2)	2 (3.8)
Expert						
Type 1	1 (100)		1(100)			
Type 2A	71(100)	2 (2.8)	66(93.0)	1 (1.4)	2 (2.8)	
Type 2B	51(100)		2 (3.9)	34(66.7)	11(21.6)	4 (7.8)
Type 3	8(100)		1(12.5)	1 (12.5)	3(37.5)	3(37.5)
Total	131(100)	2 (1.5)	70(53.4)	36(27.5)	16(12.2)	7(5.3)

JNET, Japan NBI Expert Team; HP means hyperplastic lesion; SSP/SSL, sessile serrated polyps/lesions; LGD, low-grade dysplasia; HGD, high-grade dysplasia; m-SMs, intramucosal cancer or submucosal cancer with submucosal invasion depth <1000 um; SMd, cancer with submucosal invasion depth ≥1000 um).

### Diagnostic value of JNET type 2A/2B among trainee and expert endoscopists

**Table 3** presents the diagnostic value of JNET type 2A and 2B, along with a comparative analysis between trainee and expert endoscopists.

**Table 3:** Diagnostic value of BLI combined with JNET classification between trainees and experts.

JNET classification	Diagnostic value	Trainees	Experts	*P value*
JNET type 2A	Sensitivity	0.646(51/79)	0.930(66/71)	0.000^a^
	Specificity	0.635(33/52)	0.933(56/60)	0.000^a^
	PPV	0.729(51/70)	0.943(66/70)	0.001^a^
	NPV	0.541(33/61)	0.918(56/61)	0.000^a^
	Accuracy	0.641(84/131)	0.931(122/131)	0.000^a^
JNET type 2B	Sensitivity	0.596(31/52)	0.882(45/51)	0.001^a^
	Specificity	0.734(58/79)	0.913(73/80)	0.003^a^
	PPV	0.596(31/52)	0.865(45/52)	0.002^a^
	NPV	0.734(58/79)	0.924(73/79)	0.002^a^
	Accuracy	0.679(89/131)	0.901(118/131)	0.000^a^

a Pearson's chi-square test; JNET, Japan NBI Expert Team.

For colorectal sessile lesions identified as JNET type 2A, trainee and expert endoscopists demonstrated diagnostic accuracies of 64.1% and 93.1% (P= 0.000) respectively, in predicting low-grade dysplasia (LGD) (**Table 3**). The sensitivity is 64.6% and 93.0% (P= 0. 0.000), while the specificity is 63.5% and 93.3% (P= 0.000), the PPV is 72.9% and 94.3% (P= 0.001), and the NPV is 54.1% and 91.8% (P = 0.000) respectively.

In the cases of lesions identified as JNET type 2B, trainee and expert endoscopists showed accuracies of 67.9% and 90.1% (P=0.000) in predicting HGD or m-SMs (**Table 3**). Sensitivity was 59.6% and 88.2% (P=0.001), and specificity was 73.4% and 91.3% (P = 0.003) respectively. The PPV was 59.6% and 86.5% (P = 0.002), and NPV was 73.4% and 92.4% (P= 0.002) respectively.

The interobserver agreement between expert and trainee endoscopists showed significant differences (experts: κ=0.862 for JNET 2A, κ=0.792 for JNET 2B; trainees: κ=0.272 for JNET 2A, κ=0.330 for JNET 2B; p<0.001). Used weighted kappa analysis (experts: κ=0.82 vs. trainees: κ=0.31, p<0.001) and Bonferroni correction to account for sample size differences.

### Inconsistencies in JNET identification between trainee and expert endoscopists

**Table 4** revealed that the total inconsistency rate of JNET classification between expert and trainee endoscopists was 32.8%. For trainee endoscopists’ identification of Type 2A, 69.6% (55/79) of the diagnoses were consistent with expert endoscopists, 1.3% (1/79) showed lower identification, and 29.1% (23/79) showed higher identification compared to expert endoscopists. For Type 2B identification by trainee endoscopists, 63.5% (33/52) remained unchanged, 30.8% (16/52) showed lower identification, and 5.8% (3/52) showed higher identification compared to expert endoscopists.

**Table 4:** The identification of JNET classification by experts and trainees.

Trainees	N (%)	Experts		
		Unchanged	Lower classified	Higher classified
JNET type 2A	79 (100)	55 (69.6)	1 (1.3)	23 (29.1)
JNET type 2B	52 (100)	33 (63.5)	16 (30.8)	3 (5.8)
Total	131 (100)	88 (67.2)	17 (13.0)	26 (19.8)

The *P* = 0.000, kappa = 0.367, n/N = 43/131=32.8%.

## Discussion

Differentiating malignant or premalignant colorectal lesions from benign ones is essential for determining the appropriate resection strategy. The Narrow Band Imaging (NBI)-combined JNET classification includes four types based on surface and vessel patterns to evaluate colorectal lesions: types 1, 2A, 2B, and 3. Each of the four types is intended to suggest the most likely histology (12, 14, 19). BLI, a newly developed image system for image-enhanced endoscopy using laser light, has improved the visibility and detection rates of colorectal lesions (20). Although the JNET classification was initially utilized with the NBI mode, it can also be applied to the BLI mode, with research showing high consistency between the reports of NBI and BLI magnification (21).

**Figure 2:**
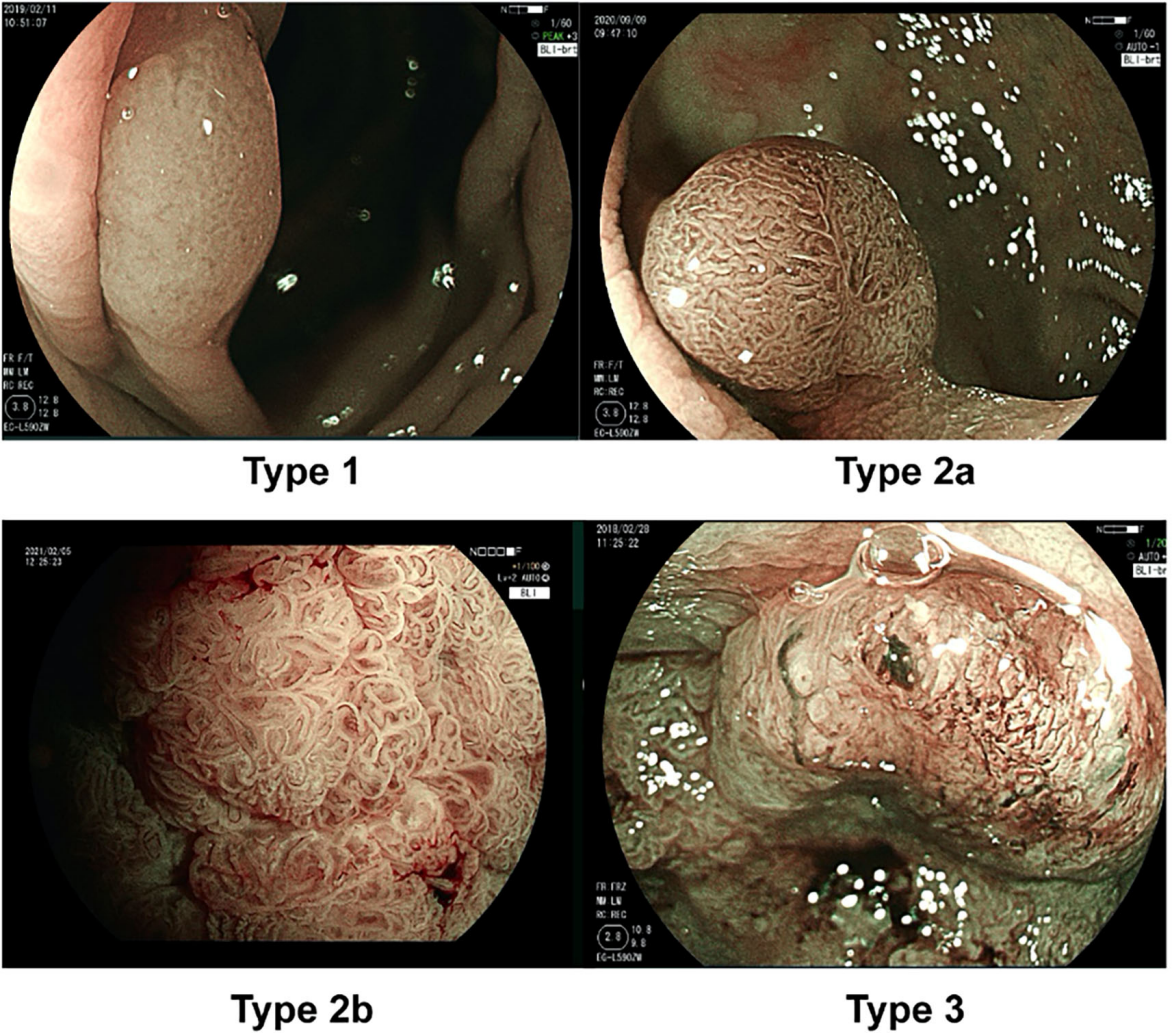
JNET classification. The characteristics of Type 1 are an invisible vessel pattern and dark or white spots on the surface. The characteristics of Type 2A are regular vessel patterns, such as regular caliber or distribution, and a regular surface pattern. The characteristics of Type 2B are an irregular vessel pattern, such as variable caliber or an irregular distribution, and an irregular or obscure surface pattern. The characteristics of Type 3 are loose vessel areas or interruptions with thick vessels and an amorphous surface pattern.

The diagnostic evaluation of colorectal sessile lesions has been significantly advanced by the integration of Blue Laser Imaging (BLI) with the JNET classification system, particularly in the hands of expert endoscopists (**Figure 2**) This study highlights the technical superiority of BLI in mucosal surface visualization, a capability that complements the vascular pattern optimization of NBI. While NBI has long been a cornerstone in endoscopic characterization, BLI’s ability to delineate subtle mucosal features—such as the cloud-like surfaces of sessile serrated lesions (SSLs) or the crypt architecture of hyperplastic polyps—provides a critical advantage for non-depressed lesions. Higurashi et al.demonstrated that BLI maintains diagnostic accuracy comparable to NBI while excelling in scenarios requiring mucosal detail (21), a finding corroborated by our results where experts achieved sensitivity and specificity exceeding 90% for both JNET type 2A (low-grade dysplasia, LGD) and type 2B (high-grade dysplasia/superficial submucosal invasion, HGD/m-SMs). This precision not only reduces unnecessary biopsies but also refines therapeutic decision-making, enabling clear stratification between lesions to decide whether to choose endoscopic resection (LGD/HGD) or surgical intervention (deep submucosal invasion, SM-d).

SSLs are a type of precancerous growth that can develop into colorectal cancer if not detected early. However, studies show significant differences in how often SSLs are found across regions. For example, in Asian countries, SSLs are detected in about 4% of patients (11), compared to 6% in younger populations in Western countries (22). Several factors are of concern relating to the differences. Doctors in some regions may not receive enough training to recognize SSLs, which are flatter and less obvious than other types of polyps. Advanced tools like BLI, which are better at detecting SSLs, may not be widely available in all hospitals. Diet, environmental factors, or genetic differences might influence how often SSLs develop in certain populations. A recent review emphasized the importance of tracking SSL detection rates (SSLDR) as a quality measure, similar to how doctors already track polyp detection rates (11). Improving SSLDR could lead to earlier detection and prevention of more cancers. Our study supports this idea—BLI’s ability to highlight subtle mucosal details makes it a powerful tool for improving SSL detection, especially in regions where these lesions are often missed.

In our Chinese cohort, 88.2% of JNET type 2B lesions were classified as HGD/m-SMs, prominently contrasting with Japanese data from Kobayashi et al. (16), where only 25.6% of JNET 2B lesions strictly met HGD/m-SMs criteria. Even when including misclassified T1b lesions, the proportion rose to just 46.7% in the Japanese cohort. This distinction likely comes from a confluence of biological and clinical factors. Apart from ethic variations in serrated pathway progression, differences in clinical practice, such as the conservative preoperative exclusion of SM-d lesions by Chinese endoscopists, may skew JNET 2B classifications toward less invasive pathologies. These findings underscore the necessity of population-specific adaptations for endoscopic classification systems, particularly in genetically diverse regions like China, where molecular heterogeneity demands tailored diagnostic frameworks.

The distinct diagnostic performance between expert and trainee endoscopists further underscores the complexity of BLI-JNET interpretation. Expert endoscopists demonstrated remarkable accuracy (93.1% for JNET 2A, 90.1% for JNET 2B), whereas trainees lagged significantly (64.1% and 67.9%, respectively). Trainee errors were multifaceted: overestimation of JNET 2A lesions (29.1% misclassified as high-grade pathologies) likely arose from difficulties in distinguishing benign mucosal patterns (e.g., regular crypt openings) from dysplastic irregularities, while underestimation of JNET 2B lesions (30.8%) reflected challenges in recognizing microvascular distortions indicative of submucosal invasion. While Kang et al. (23) reported rapid training sufficiency in diagnosing small polyps using i-scan optical enhancement (OE), our findings highlight challenges in BLI-JNET for larger, morphologically complex sessile lesions. The technical complexity of laser-enhanced mucosal analysis in managing invasive lesions likely explain this difference. Thus, structured training programs for advanced interpretive skills are required for an endoscopist before independent practice.

The principles underlying BLI-JNET extend beyond this specific technology (24). The prospective study utilizing i-scan OE with the NICE classification achieved comparable diagnostic accuracy for small polyps, illustrating the universal applicability of optical enhancement strategies (23, 24). These modalities—whether prioritizing mucosal surface detail (BLI) or vascular clarity (NBI/i-scan OE)—are complementary rather than competitive. Future innovations should explore hybrid systems that integrate these modalities, or investigate the mechanisms between endoscopic phenotypes (e.g., JNET types) and genomic markers (4).

Despite these advancements, our study has limitations. The retrospective design and exclusion of surgically resected JNET type 3 lesions (SM-d) may underestimate diagnostic challenges for advanced cancers. Static image analysis, while standardized, cannot replicate the dynamic assessment of lesion mobility or mucosal stiffness critical to real-time endoscopy. Prospective multicenter trials evaluating next-generation BLI systems (e.g., BLI-bright) across different populations are essential to validate and refine these findings.

In conclusion, BLI-JNET is a valuable tool for diagnosing colorectal lesions, but its success depends on some key factors. Trainee endoscopists need structured programs to master BLI interpretation. Classification systems must account for local differences in biology and medical practices. By improving training and adopting quality measures like SSLDR, doctors worldwide can catch more precancerous lesions early, ultimately saving lives through timely intervention.

## Data Availability

The raw data supporting the conclusions of this article will be made available by the authors, without undue reservation.
